# The heterogeneous relationship between public health expenditures and household medical expenditures: evidence from relative poverty households in China

**DOI:** 10.3389/fpubh.2023.1195223

**Published:** 2023-08-24

**Authors:** Zhenyu Li, Xingzhang Yuan, Zhao Zhang, Yuning Chu

**Affiliations:** ^1^School of Economics, Qingdao University, Qingdao, China; ^2^School of Economics and Finance, Xi’an Jiaotong University, Xi’an, China; ^3^Department of Nutrition, The Affiliated Hospital of Qingdao University, Qingdao, China

**Keywords:** public health expenditures, household medical expenditures, relative poverty, crowding-in effect, heterogeneity

## Abstract

Exploring the heterogeneous relationship between public health expenditures and household medical expenditures from the perspective of relative poverty has positive significance for improving the ability of relative poverty households to cope with the risk of large rigid expenditures and optimizing the public health resource allocation. This paper uses the China Family Panel Studies (CFPS) from 2016 to 2020 to identify relative poverty standard from the perspective of medical needs, analyzes the impact of public health expenditures on medical expenditures of different household types, and evaluates the effect of public health expenditures on ensuring the medical needs of relative poverty households. The panel threshold regression result shows that 19.36% of the provinces *per capita* disposable where the household is located is the identification standard of relative poverty households. Public health expenditures have a crowding-in effect on household medical expenditures and have a stronger impact on relative poverty households, an effect that is also confirmed by two-stage least squares regression. In addition, ensuring household medical needs through public health expenditures focuses on the level of basic medical needs, and the role of household healthcare expenditures that reflects high-level medical needs is not obvious. In the future, the government should establish a monitoring mechanism for relative poverty households, ensure the basic medical needs of relative poverty households, and clarify the heterogeneity among different types of households, provide targeted public health services.

## Introduction

In recent years, with the increasing awareness of health investment, household medical expenditures, as an important component of household consumption, have grown rapidly. Data shows that Chinese household medical expenditures have increased from 4968.11 yuan in 2014 to 6236.9 yuan in 2020.^1^ At the same time, the expenditure gap between households is also widening. Its growth is nearly three times compared with the absolute growth, which is particularly evident among households with different incomes and consumption needs. At present, the focus of poverty governance in China has shifted from “absolute poverty” to “relative poverty”.^2^ Unlike absolute poverty, relative poverty reflects “relative deprivation” (1). It manifests as unequal access to public services by households, as well as a low level of social security, such as healthcare, education, and pensions (2). Compared with ordinary households, relative poverty households have a heavier healthcare burden, and are more vulnerable to exogenous shocks and return to poverty (3). Therefore, how to meet the medical needs of relative poverty households and improve their ability to cope with rigid expenditure risks will be an important guarantee for achieving relative poverty governance. Public health expenditures, as an effective means for the government to provide public medical services and meet the basic medical needs of the public, will also play a crucial role in relative poverty governance (4).

However, on the one hand, the identification standard of relative poverty households is not clear, and the relatively poverty group cannot be identified from the perspective of household medical needs. On the other hand, although public health expenditure aims to ease the healthcare burden of households (5), the heterogeneity effect of public health expenditures on household medical expenditures of relative poverty and ordinary households has not been empirically tested. The above reasons make it difficult to evaluate the effectiveness of public health expenditures on meeting household medical needs at present.

On the basis of the above background, this paper takes Chinese households as a research sample and uses the China Family Panel Studies (CFPS) from 2016 to 2020 to address two issues: Firstly, to determine the relative poverty standard and identify relative poverty groups from the perspective of household medical needs. Secondly, to explore public health expenditures, their relationship with household medical expenditures and the heterogeneity among groups, and then evaluate their effect on ensuring the relative poverty household medical needs. The results of this paper will help to understand the current relative poverty situation and the effectiveness of public health expenditure in meeting the relative poverty household medical needs. It is of great significance for improving the construction of public health service systems and governing relative poverty.

## Literature review

### Relative poverty measurements and governance

There are obvious differences between relative poverty and absolute poverty in terms of connotation, measurement standards and governance methods. At present, researches on relative poverty are still insufficient, especially regarding the identification of poverty objects and the determination of measurement standards. The common international identification methods and measurement standards of relative poverty are still similar to absolute poverty, which reflects the characteristics of monetization dimension of income and is defined as a certain percentage number lower than the median income, i.e., the relative income standard (6, 7). Referring to this practice, some studies further propose calculating the relative poverty standard using the average income level of residents (8) or a certain proportion of the median income of urban and rural residents (9). Some researches have proposed that the coverage of social assistance can be used as a reference for determining the proportion of the relative poverty population (10). Similarly, Au (11) uses a cost-of-living approach to measure relative poverty, where the poverty line is defined as the cost of essential goods and services. Although similar measures are easy to apply, they cannot reflect the unique demand (or expenditure) dimension of relative poverty compared with absolute poverty, leading to a serious underestimation of the depth of poverty. Recently, some scholars have called for the establishment of a relative poverty identification and measurement system that considers income type and demand type (12) and have proposed a plan based on demand (13). For example, He and Zhu (14) appeal for labor mobility as a measure to contain relative poverty. Zhang and Su (15) state that unfair allocation of social resources and the household registration system is partially responsible for the existence of relative poverty in China. However, no specific identification standards and quantitative measures have been established.

In recent years, scholars have analyzed the causes of relative poverty and countermeasures from an institutional perspective. Ravallion (16) concludes that the substantial difference in basic public services such as medical care and education constitutes national inequality, subsequently forming the problem of relative poverty that exists today. In the process of relative poverty governance, health human capital (17), educational human capital (18) and social capital (19) are replacing traditional capital, such as local economic development and infrastructure improvements, and have become the main factors in reducing poverty. Therefore, to safeguard publics’ right to subsistence and development, it is necessary to establish a demand-oriented security system and address expenditures such as household medical care, education, and housing that have important impacts on publics’ right to subsistence and development (20). The fair public policy system for the relative poverty further emphasizes the equalization of basic public services and maintenance of social equity (21).

### The heterogeneous relationship between public health expenditures and household medical expenditures

As a type of financial expenditure, the role of public health expenditures on household consumption is controversial in theory, and the relationship between public health expenditures and household medical expenditures has not yet been determined empirically.

Some studies support the idea of a “crowding-out effect” between the two types of spending. This perspective emphasizes the impact of public health expenditures on the supply side of medical services and suggests that government public health expenditures reflect public finance support for medical and healthcare and that an increase in government investment in public health can directly or indirectly reduce personal medical expenditures (22, 23). Additionally, greater government investment in basic medical and health services can improve public health facilities and improve the level of medical security, and the popularization of basic medical and health services can improve individuals’ ability to prevent diseases, reduce individual morbidity rates, and reduce individual medical expenses. Many scholars have found through empirical research that public health expenditures are an important factor affecting personal medical expenditures and verified that public health expenditures have a crowding-out effect on personal medical expenditures (24, 25).

Other studies support the idea of a “crowding-in effect” between the two types of spending. This perspective emphasizes the impact of public health expenditures on the demand side of medical services and suggests that an increase in public health expenditures improves the level of government medical security, which in turn increases the budget constraints of personal medical consumption, releases part of the personal consumption demand for medical services, and ultimately improves the level of personal medical spending. For example, Long et al. (26) conducted a study on the effectiveness of China’s 2000–2010 healthcare system reforms and found that public health expenditures had a significant “crowding-in” effect on personal medical expenditures and the effect was more pronounced in rural and underdeveloped areas. Similarly, Dieleman et al. (27), by examining changes in public health spending and private health spending in the United States, found that public health spending significantly contributed to private health spending. From the perspective of the economic effects of public health spending, some scholars have also found that public health spending stimulates medical consumption while stimulating personal nonmedical consumption (28).

Due to the different directions of public health spending on the supply and demand sides, the final impact on household medical spending will depend on the magnitude of the positive and negative effects. While, some studies have noted that there is also heterogeneity in the final impact influenced by certain features. For example, business cycle (29), public expenditure content (30), household welfare situation (31), etc. Especially, household income is an important feature. As income increase, the promoting effect of public health expenditure on household consumption expenditure may gradually weaken (32, 33).

### Evaluation of existing research and contributions of this paper

The existing studies provide a useful reference and inspiration for this paper. From the research perspective, existing studies confirm that structural inequalities in household medical expenditures and “growth differentiation” exacerbate the cumulative health disadvantages of relative poverty households, leading to the continuous expansion of the human capital gap between groups, which reflect the basic logic of relative poverty caused by household medical needs. However, few studies take this mechanism into account when analyzing relative poverty. This paper identifies the relative poverty standard from the perspective of household medical needs, considering the possibility of relative poverty caused by differences in medical needs between households. From the research content, the existing research on the relationship between public health expenditures and household medical expenditures has not reached a consistent conclusion. When examining the relationship between the two, the impact of public health expenditure on the medical needs of different household types has been ignored. This paper further explores the heterogeneity in the effects of public health expenditures across different types of households, which is an important mechanism for the governance of relative poverty through public health expenditures. From the research methods, there are many qualitative studies and few quantitative studies on the governance of relative poverty, and most of them focus on the policy support of public service supply. This paper uses the panel threshold model, fixed effect model, two-stage least squares method (2SLS) model and other research methods to obtain quantitative results on related issues to improve the pertinence of relative poverty governance strategies.

## Research design

### Theoretical basis and research hypothesis

**Figure 1:**
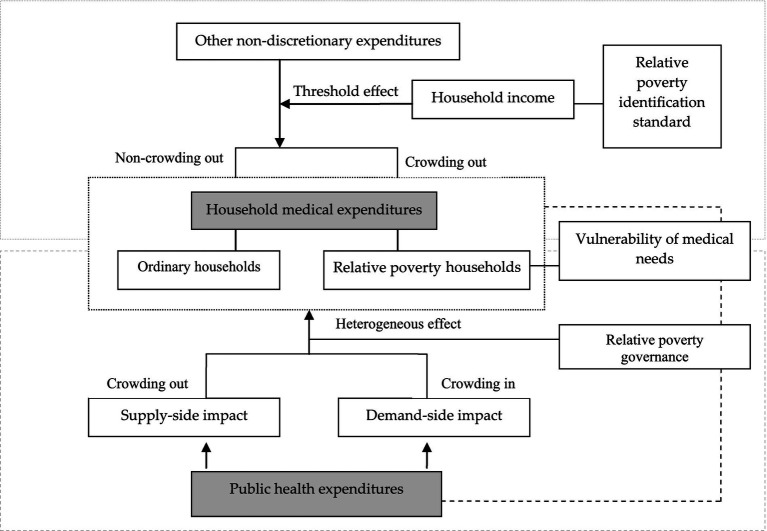
The research roadmap.

Figure 1 is the research roadmap for this paper, which mainly involves two issues: first, measuring relative poverty standards and identifying relative poverty households from the perspective of household medical needs; Second, to explore the heterogeneous relationship between public health expenditure and medical expenditure of different types of households, and to evaluate its effectiveness in ensuring the medical needs of relative poverty households.

For issue 1, human capital theory (34) proposes that human capital is the sum of the value of workers’ knowledge, health status, etc., and can improve by expanding investments in education, healthcare, etc. The household investment model proposed by Becker (35) provides a theoretical framework for studying household medical expenditure decisions. Under the household code of conduct in pursuit of utility maximization, household medical expenses are constrained by various factors, such as household background, household socioeconomic status, and medical service prices, which are the result of a series of external factors. Among the many factors, household income is the most prominent. Low-income households are more vulnerable to household medical expenditures due to risk aversion or borrowing constraints and are at higher risk of catastrophic medical expenditures. The uncertainty of household income and uncertainty of future returns of health investments have negative impacts on household medical expenditures, and this effect is more pronounced for low-income households (36).

Compared with absolute poverty, relative poverty involves not only household food expenditures but also household development expenditures (or household large rigid expenditures) such as healthcare, education, and housing. On the basis of the household investment model, this paper argues that due to household consumption demand preferences and liquidity constraints, the large rigid expenditures of households on certain aspects in a certain period may far exceed household disposable income in the same period, thereby affecting other household investment decisions. When the *per capita* disposable income of a household is low, especially for relative poverty households, to meet the necessary expenditure needs, the household may maintain a large amount of rigid expenditures (such as household education expenditures), which may crowd out the household’s medical expenditures in the same period, indicating that the two are negatively correlated. At this time, households are highly vulnerable and at potential risk of not meeting developmental consumption needs, especially medical needs. When the *per capita* disposable income of a household reaches a certain level, with the increase in capital investment demand and residents’ active health awareness, the crowding out effect between the two types of expenditure may weaken or disappear, and a parallel growth relationship will appear.

Therefore, we propose H1: there is a certain income threshold value between household medical expenses and other rigid household expenditures. Above and below this income threshold value, the correlation between the two types of expenditures in the same period is completely different, and this threshold value can be used as an identification standard for relative poverty households from the perspective of household medical needs.

For issue 2, because household medical expenditures are the main component of large rigid household expenditures, these expenses are an important measure of household medical demand. Combined with theoretical analyses and existing research conclusions, public health expenditures on the supply side can improve the availability of medical and health resources, and the impact of these expenditures on household medical expenditures manifests as a crowding-out effect; in contrast, public health expenditures on the demand side can improve residents’ medical treatment and meet residents’ medical needs, and the impact on household medical expenses manifests as a crowding-in effect. In reality, since China launched its “new medical reform” policy in 2009,^3^ the total amount of financial investment in primary medical and health services has increased rapidly, the serious shortage of supply capacity in the primary medical and health service system has been reversed, and the impact of public health expenditures on the supply side has been alleviated. Overall, the impact of public health spending on household medical spending may manifest as a crowding-in effect.

However, due to the vulnerability of relative poverty households in terms of living security, there is still a large gap between their expected medical needs and affordable medical needs, and the gap in medical consumption needs is larger for relative poverty households than for ordinary households. The government’s investment in basic medical services and medical security has improved accessibility to medical services, lowered the price of medicines, and to a certain extent lifted the budget constraints of low-income groups, greatly stimulating their willingness to consume. In contrast, ordinary households, especially the high-income households, have a strong ability to pay. On the premise that the needs of basic medical services are fully met, these households consume high-quality and high-level medical services. The basic medical services and medical security provided by the government finance may not be very attractive for their consumption.

Therefore, we propose H2: generally, there is a crowding-in effect between public health expenditures and household medical expenditures, but this effect is heterogeneous between ordinary households and relative poverty households, and the crowding-in effect on relative poverty households may be more obvious.

### Methods and variable selection

To test H1, identifying possible income thresholds is a necessary part of determining the identification standard of relative poverty households. Hansen (37) believes that the threshold effect between economic factors can be analyzed using the threshold panel model. Therefore, based on the theory of human capital, this paper uses the panel threshold regression model to systematically analyze the changing relationship between the household medical expenditures and another important household large rigid expenditure, household education expenditures with different income levels in the same period, and then determine the identification standard and identify relative poverty households from the perspective of household medical needs.

The model can be expressed as:


(Model 1) $$\begin{array}{l} {\mathrm{ME}}_{it}=\beta_{1}{\mathrm{EDU}}_{it}\cdot l\;\left(I_{it}\leq \gamma \right)+\beta_{2}{\mathrm{EDU}}_{it}\cdot \\ \;\;\;\;\;\;\;\;\;\;\;\;\;\;\;\;\;\;l\;\left(I_{it}>\gamma \right)+\beta X+\mu_{i}+\varepsilon_{it} \end{array}$$

*l*(·) is an indicative function, and *I* is a threshold variable, which is determined by the ratio of the *per capita* disposable income of household *i* in year *t* to one-third of the *per capita* disposable income of the province where the household is located. The reasons for choosing this indicator are as follows. First, the selection of threshold variables should reflect the consumption demand and budget constraints faced by households, and reflect the different changing relationships between the explanatory variables and the explained variables above and below the threshold value; therefore, the threshold variable is constructed using the *per capita* income of households. Second, two dimensions of income and expenditure (or demand) included in the concept of relative poverty are considered in this indicator. It is reasonable to assume that households with lower income levels are more vulnerable to no meeting their medical needs than are higher income households. Additionally, to take into account the other rigid expenditure burdens of the household to maintain basic life, when the threshold variable is constructed, one-third of the *per capita* disposable income of each province is used as the standard to delineate the high-probability groups that are prone to falling into relative poverty,^4^ the regression samples were screened using this standard. On this basis, relative poverty households are identified from the perspective of medical needs. Finally, to enhance the relevance of the measurement standard, the use of relative indicator can provide different relative poverty identification standards in different provinces. *γ* is the threshold value to be measured in this paper. There are different correlations between EDU*_it_* and ME*_it_* in the two cases of *I_it_ ≤ γ* and *I_it_ > γ*. *β*_1_ and *β*_2_ represent the influence coefficients between household medical expenditures and education expenditures above and below the threshold *γ*, respectively. If there is a threshold γ^*^ such that *β*_1_ < 0 and *β*_2_ > 0, then γ^*^ is the identification standard of relative poverty households from the perspective of household medical needs.

The explained variable *ME_it_* used in this paper is the sum of the medical expenditures of household *i* in year *t*.^5^ The explanatory variable EDU*_it_* is the sum of the education expenditures of household *i* in year *t*. The reason for choosing household education expenditures is mainly because existing researches have merged four types of expenditures: education, medical, housing, and older adult care into large rigid household expenditures from the perspective of Chinese household expenditure (38). In these four types of expenditures, the two large rigid expenditures, i.e., household housing and older adult care, are more susceptible to the influence of macro policies and greater uncertainty. Additionally, the other two large rigid expenditures are difficult to measure and obtain data in existing databases. Both the explanatory variables and explained variables are in logarithmic form. Based on available data, referring to related studies on factors that influence household spending decisions (39), *X* is a series of control variables. Such as age, gender, health level, smoking, marital, medical insurance, household registration, household size. *μ_i_* is the individual effect, *ε_it_* is the disturbance term.

To test H2, the following fixed effects model is constructed as benchmark regression. Furthermore, the samples are grouped based on the relative poverty identification standard above. The heterogeneity of the impact of public health expenditures on different types of household medical expenditures is examined. The specific standard for sample grouping is determined using the threshold value γ^*^ in Model 1.

The benchmark regression model is as follows:


(Model 2) $${\mathrm{ME}}_{iyt}=\delta_{1}{\mathrm{PHE}}_{iyt}+\delta X+\mu_{y}+\varepsilon_{iyt}$$

The explained variable ME*_iyt_* is still the medical expenditures of household *i* in year *t*, and the core explanatory variable PHE*_iyt_* is the public health expenditure of province *y* where household *i* is located in year *t*, measured by “*per capita* government health expenditures.” Both explanatory variables and explained variables are in logarithmic form. *δ*_1_ represents the impact of public health expenditures on household medical expenditures. Control variables *X* includes all the control variables in Model 1 in addition to macro indicators such as regional GDP *per capita* and other indicators that can represent the local medical service level of province *y*. *μ_y_* is the regional effect, *ε_iyt_* is the disturbance term.

Furthermore, this paper introduces “the sum of healthcare expenditures of household *i* in the past 12 months” in Model 2 as a substitute variable for the explained variable ME*_iyt_*. In the database, household healthcare expenditures measure the consumption of fitness exercise and purchasing related products, equipment, healthcare products, etc. Compared with household medical expenditures, household healthcare expenditures can reflect a household’s medical and health needs at a higher level.

Finally, to eliminate possible endogeneity in the model, this paper uses the two-stage least squares method (2SLS) to estimate the relationship between public health expenditure and household medical expenditure. Due to the strong subjective purpose of some macro policies, they can also affect the current household medical expenditure while affecting the public health expenditure. With reference to similar researches (40, 41), this paper takes the “*per capita* government health expenditures of each province in the previous year” as the Instrumental variables estimation. There is reason to believe that the level of government health investment during a certain period is continuous, and there is a high correlation between *per capita* government health expenditure in adjacent periods. And because it is a predetermined variable that has already occurred in the previous period, and its value is fixed from the perspective of the current period, it can be considered unrelated to the disturbance term in the current period. Model 3 and Model 4 are the first and second stage estimation models of 2SLS, respectively, and focus on the sign and significance of *θ*_1_ in Model 4.


(Model 3) $${\mathrm{PHE}}_{iyt}=\eta_{1}{\mathrm{PYPHE}}_{iyt}+\eta X+\mu_{y}+\varepsilon_{iyt}$$

(Model 4) $${\mathrm{ME}}_{iyt}=\theta_{1}{\bar{\mathrm{PHE}}}_{iyt}+\theta X+\mu_{y}+\varepsilon_{iyt}$$

### Dataset

This paper takes Chinese households as the research sample. The micro data used is derived from panel data from the China Family Panel Studies (CFPS), which was released by the China Social Science Survey Center of Peking University in 2016, 2018, and 2020. The main reason for choosing this database is that it involves micro level data from individuals, households, and communities, providing rich information about public health conditions and residents’ consumption, and vividly depicting changes in health and social welfare. The macro data on public health expenditure and so on in various provinces are from the China Health Statistical Yearbook and China Statistical Yearbook of each year. During the analysis process, expenditure variables were deflated using 2016 as the benchmark, and values were assigned at the micro level based on the province where the household locates. For the processing of outliers, this article intercepts the data between the upper and lower 1% quantiles of the relevant variables for sampling.

## Results

### Descriptive statistics

Table 1 shows the results of the data descriptive analysis. From the perspective of household level, the *per capita* disposable income of Chinese households continued to grow from 2016 to 2020. As two important large rigid expenditures, household expenditures on medical and education accounted for 8.52% and 6.65% of total household disposable income in 2020. If only the income dimension relative poverty sample is considered, the total proportion of the two types of expenditures is close to 50%. The higher proportion of two expenditures also lays the foundation for this paper to determine the relative poverty standard. In addition, the consumption gap between households is continuing to widen. Due to the impact of household essential medical expenses and severe illness shocks, the standard deviation of household medical expenses is greater, and household education expenditures are more rigid. In addition, the noteworthy indicator is “medical insurance.” The average value in 2016 was 0.93, indicating that 93% of the samples have medical insurance, reflecting the significant increase in medical insurance coverage since the implementation of China’s “new medical reform” policy, which also provides protection for the release of household medical needs. From the perspective of macro data, *per capita* government health expenditure continued to increase from 2016 to 2020, but the investment gap between provinces continued to widen. The *per capita* government health expenditure has increased by about 493 yuan. Government investment is playing an increasingly important role in the entire health system. From the perspective of medical service level, the number of hospital personnel per 10,000 people and the number of beds in medical institutions per 10,000 people have also increased significantly, but the standard deviation between provinces has also expanded, and the horizontal fairness of public health services needs to be strengthened.

**Table 1:** Descriptive statistics.

Year	Variables		
	2016	2018	2020
	Mean (standard deviation)	Mean (standard deviation)	Mean (standard deviation)
Household medical expenses (yuan)	5350.19 (13438.54)	5828.46 (15170.11)	6236.90 (16887.40)
Household education expenditure (yuan)	4459.66 (6224.68)	5163.86 (7020.78)	5529.73 (7413.57)
*Per capita* disposable income of households (yuan)	22109.13 (31554.08)	25959.28 (35409.36)	27682.28 (38981.32)
Age	47.24 (14.02)	49.15 (14.08)	51.15 (14.08)
Gender (male = 1, female = 0)	0.51 (0.49)	0.52 (0.49)	0.52 (0.49)
Health level (from 0 to 5, the higher the value, the worse the health level)	3.04 (1.20)	3.08 (1.20)	3.04 (1.21)
Marital status (married = 1, unmarried = 0)	0.82 (0.35)	0.82 (0.36)	0.82 (0.35)
Household register (rural = 1, urban = 0)	0.73 (0.42)	0.72 (0.44)	0.72 (0.44)
Household size	3.69 (1.89)	3.55 (1.88)	3.71 (1.88)
Medical insurance (yes = 1, no = 0)	0.93 (0.25)	0.93 (0.24)	0.93 (0.26)
Smoking (yes = 1, no = 0)	0.28 (0.45)	0.29 (0.45)	0.28 (0.45)
*Per capita* public health expenditure (yuan)	972.42 (264.46)	1121.66 (317.49)	1465.18 (410.69)
*Per capita* GDP (yuan)	52259.73 (23782.56)	60544.65 (27505.64)	65365.45 (30028.97)
Number of hospital personnel per 10,000 people	82.73 (12.85)	90.54 (13.42)	95.54 (14.51)
Number of beds in medical institutions per 10,000 people	53.59 (6.91)	59.88 (8.09)	64.84 (8.74)

### Relative poverty standard measurement from the perspective of household medical needs

Table 2 shows the regression results for the panel threshold model. The regression process is divided into two steps. The first step is the threshold effect test, that is, the number of thresholds is determined, and then, the model form is determined. The second step is a test of the estimated threshold value. In the first step, Model 1 is estimated under the settings of a single threshold and double threshold. The obtained *F* statistics are shown in Table 2. The results indicate that the *F*-statistic of the single-threshold test is 19.77 and that the single-threshold effect is rejected at the 1% significance level. The *F*-statistic of the double-threshold test is 6.20 and not significant, i.e., no threshold effect. Therefore, there is a single threshold in the model setting. Regarding the 95% confidence interval threshold, the estimated value of the single threshold is 0.5807, that is, the *per capita* disposable income of the household accounts for 58.07% of one-third of the *per capita* disposable income of the province where the household is located. In the second step of the threshold test, the threshold value is estimated. The regression results for each variable are shown in Table 2. Because a single threshold is accepted as the setting, the regression results in the second column of Table 2 are pertinent. The relationship between household education expenditures and medical expenditures is significantly different around the first threshold *γ*_1_. When the threshold variable value is less than *γ*_1_, there is a significant negative relationship between the two expenditures at the 1% significance level. When the threshold variable value is more than *γ*_1_, there is a significant positive relationship between the two expenditures at the 1% significance level. The crowding out effect between the two disappears, and parallel growth characteristics appear instead.

**Table 2:** Panel threshold regression results.

	Single threshold	Double threshold
Threshold value	*γ*_1_ = 0.5807	*γ*_1_ = 0.5807
		*γ*_2_ = 0.1321
*F*	19.77^***^	6.20
Household medical expenses (*I_it_ < γ*_1_)	−0.0615^***^ (0.0223)	−0.0709^***^ (0.0205)
Household medical expenses (*γ*_1_ *≤ I_it_ ≤ γ*_2_)	0.0714^***^ (0.0218)	0.0065 (0.225)
Household medical expenses (*I_it_ > γ*_2_)		0.0711^***^ (0.0218)
Age	−0.0956^***^ (0.0237)	−0.0982^***^ (0.0237)
Gender	0.3719 (1.9528)	0.3830 (1.9522)
Health level	0.1330^***^ (0.0510)	0.1346^***^ (0.0510)
Marital status	0.7381^**^ (0.3628)	0.7251^**^ (0.3628)
Household register	0.0090 (0.0315)	0.0094 (0.0315)
Household size	0.0756 (0.0529)	0.0795 (0.0529)
Medical insurance	0.4801^***^ (0.1780)	0.4851^***^ (0.1780)
Smoking	−0.1571 (0.2442)	−0.1589 (0.2441)
Intercept term	10.3596^***^ (1.7323)	10.4903^***^ (1.7336)
*N*	3,189	3,189
Adjusted *R*^2^	0.2015	0.2026

(1) The brackets are heteroscedasticity robust standard errors. (2) ^*^, ^**^ and ^***^ are statistically significant at the significance levels of 10%, 5% and 1%, respectively.

**Figure 2:**
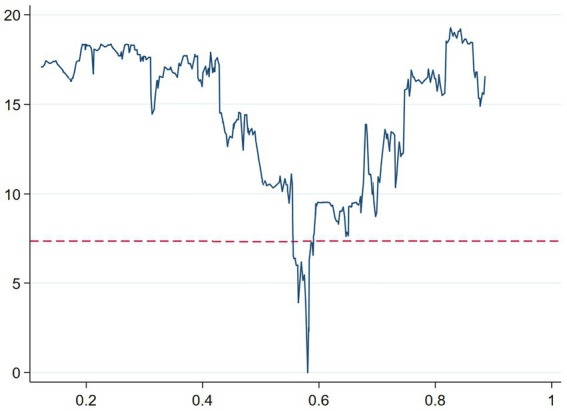
Threshold effect test LR function diagram.

To test whether the estimated threshold value *γ*_1_ is consistent with the true value, we drew a threshold effect test LR function diagram. The dashed line in Figure 2 represents a confidence value of 7.35 at the 5% significance level, while the solid line represents the likelihood ratio statistic LR of the threshold variable. When LR is equal to 0, the threshold estimate value can be obtained. In Figure 2, it can be seen that the threshold value *γ*_1_ obtained in this paper exactly matches the corresponding value of the lowest point of the solid line. At the same time, the areas below the intersection of the solid and dashed lines fall within the 95% confidence interval of *γ*_1_, indicating that the estimated threshold value *γ*_1_ is consistent with the true value.

Therefore, we propose that the threshold variable should be 0.5807. Because households below this standard cannot meet their medical needs or have rigid expenditure vulnerabilities from the perspective of medical needs, 58.07% of one-third of the province’s *per capita* disposable where the household is located should be considered as relative poverty standard under the perspective of household medical needs.^6^

Regarding the regression results for the other variables in column 2 of Table 2, we focus on two variables: the health level and medical insurance of householder. The poorer is the health level of the householder, the more household medical expenses. Due to the lower threshold of medical insurance reimbursement and the increase in the reimbursement ratio, participating in medical insurance will significantly increase household medical expenses.

### The heterogeneous relationship between public health expenditures and household medical expenditures

Table 3 shows the regression results for Model 3. For the total sample (column 2 of Table 3), an increase in public health expenditures will increase household medical expenditures at the 10% significance level, and public health expenditures have a crowding-in effect on household medical expenditures. This result indicates that with the continuous increase in Chinese government medical expenditures since the “new medical reform” policy, the supply of medical resources has increased, and the availability of medical services has improved. Additionally, government subsidies, expanding medical insurance coverage and the reimbursement ratio have lowered the threshold for seeing a doctor and eased the medical needs of households to a certain extent. The past predicament of “do not dare to seek medical treatment” caused by a household’s economic capacity limitations has improved.

**Table 3:** Fixed effects regression results.

Variables	Model					
	“Household medical expenses” as the explanatory variable			“Household healthcare expenditure” as the explanatory variable		
	All Sample	Relative poverty households (*I_it_ ≤* 0.5807)	Ordinary households (*I_it_ >* 0.5807)	All Sample	Relative poverty households (*I_it_ ≤* 0.5807)	Ordinary households (*I_it_ >* 0.5807)
*Per capita* public health expenditure	0.1727^*^ (0.1016)	0.2,606^**^ (0.1448)	0.1686 (0.1054)	−0.2418 (0.2207)	−0.1139 (0.6842)	−0.2436 (0.2216)
Age	0.0329^***^ (0.0114)	0.0424^***^ (0.0010)	0.0358^***^ (0.0121)	0.0262 (0.0295)	−0.0074 (0.0672)	0.0323 (0.0296)
Gender	0.0114 (0.3493)	0.0215 (0.4775)	0.1792 (0.3648)	0.3263 (0.6360)	0.1737 (1.8931)	0.3456 (0.6317)
Health level	0.0942^***^ (0.0093)	0.1477^***^ (0.0368)	0.0919^***^ (0.0103)	0.0564^**^ (0.0281)	−0.0790^**^ (0.0387)	0.0493^*^ (0.0284)
Marital status	0.3186^***^ (0.0569)	0.3150^***^ (0.0619)	0.2898 (0.2521)	0.0844 (0.1409)	0.0016 (0.2648)	0.1057 (0.1408)
Household register	−0.0026 (0.0044)	−0.1134^*^ (0.0673)	−0.0013 (0.0045)	−0.2055^***^ (0.0106)	−0.1405 (0.0916)	−0.1978^***^ (0.0106)
Household size	0.1185^***^ (0.0089)	0.0542 (0.0475)	0.1348^***^ (0.0099)	0.0511^*^ (0.0286)	0.0056 (0.0491)	0.0333 (0.0290)
Medical insurance	0.0598^*^ (0.0353)	0.1959^*^ (0.1152)	0.0493 (0.0387)	0.1785^*^ (0.0965)	0.1504 (0.1372)	0.1693^*^ (0.0971)
Smoking	−0.1812^***^ (0.0434)	−0.4083^**^ (0.1696)	−0.1432^***^ (0.0479)	−0.2233^*^ (0.1222)	−0.0113 (0.1750)	−0.2584^**^ (0.1245)
*Per capita* GDP	0.0038 (0.0868)	0.3426 (0.4840)	−0.0212 (0.0916)	0.4507^*^ (0.2361)	0.3384 (0.4815)	0.5067^**^ (0.2357)
Number of hospital personnel per 10,000 people	0.0066^**^ (0.0027)	0.0067 (0.0168)	0.0058^**^ (0.0029)	−0.0035 (0.0062)	−0.0033 (0.0179)	−0.0054 (0.0062)
Number of beds in medical institutions per 10,000 people	0.0088^***^ (0.0032)	0.0148 (0.0192)	0.0079^**^ (0.0034)	0.0064 (0.0077)	−0.0287 (0.0193)	0.0074 (0.0078)
Intercept term	3.5006^***^ (0.9064)	5.4826 (5.0021)	7.3455^***^ (0.9509)	1.6907 (2.301)	−3.1082 (5.0479)	1.0244 (2.2951)
Regional fixed effect	Yes	Yes	Yes	Yes	Yes	Yes
*N*	19,152	3,441	15,711	3,783	246	3,537
Adjusted *R*^2^	0.3501	0.2808	0.3402	0.2135	0.1773	0.20896
The empirical *p*-value		0.008^***^			0.226	

(1) The brackets are heteroscedasticity robust standard errors. (2) ^*^, ^**^ and ^***^ are statistically significant at the significance levels of 10%, 5% and 1%, respectively. (3) The empirical *p*-value is calculated using Fisher combination test (bootstrap 1,000 times).

The samples are grouped into relative poverty households and ordinary households from the perspective of household medical needs. The regression results (columns 3–4 in Table 3) indicate that the crowding-in effect of public health expenditures on the medical expenditures of relative poverty households is significant at the 5% level; importantly, the impact on ordinary households is small and nonsignificant. We use the Fisher combination test to perform a significance test on the differences in coefficients between two types of households. It can be seen that the empirical *p*-value obtained based on the core explanatory variable regression coefficient is 0.008, indicating that the heterogeneity of the relationship between public health expenditures and household medical expenditures is significant at the 1% significance level. This is consistent with H2. During the transition from low to high income, due to the large gap in the basic medical needs of relative poverty households, the consumption of medical services and drugs is largely constrained by income. The government’s investment in basic health services and health care coverage has eased the income constraints for relative poverty households to access health care and has a strong crowding-in effect. For ordinary households, especially high-income households, the basic medical needs have been met, and more emphasis is placed on the consumption of high-quality, high-level healthcare products and services. This cannot stimulate an increase in medical consumption, and the crowding-in effect is weak.

The regression results for other variables are not significantly different among the three samples. Taking the regression results for relative poverty households as an example, age, health level, marital status and medical insurance all significantly increase the level of household medical expenditures. The household medical expenditures of rural residents are significantly lower than that of urban residents. Participating in medical insurance significantly increases the medical expenditure level of relative poverty households; however, this variable is not significant for ordinary households. This, to some extent, illustrates the stronger impact of public health expenditures on the medical needs of relative poverty households. Among the macro variables, the number of hospital personnel and beds in medical institutions per 10,000 people has a significant positive effect in the full sample and ordinary households, the others are not significant. This finding indicates that at the household level, especially for relative poverty households, macro factors such as economic development and medical services have little impact on household medical expenditures and that the level of household medical expenditures is more limited by household income, health status and financial security.

The above perspective is supported by the regression results (columns 5–7 in Table 3) with “household healthcare expenditures” as the explained variable. The impact of public health expenditures on household healthcare expenditures is negative and not significant. This finding indicates that the increase in the supply of medical services cannot significantly effect household healthcare consumption. The protection of public health expenditures for household medical needs is more reflected in basic medical services, for the higher-level healthcare needs of households, the effect of financial expenditures is not obvious. High-level medical needs are more limited by the environment and economic development level where the household is located. The coefficient of household register and *per capita* GDP are significant.

Finally, from the regression results of the 2SLS model (columns 2–4 in Table 4), the impact of *per capita* government health expenditures on household medical expenditures is slightly larger than that calculated using Model 3 but remains stable. There is a crowding-in effect of government health expenditure on household medical expenditures, and it is stronger for relative poverty households. The Fisher combination test indicates that this difference between two groups is statistically significant at the 10% significance level. The chi^2^ value of the *C* statistic of the endogeneity test and the *F* value of the Cragg–Donald Wald statistic of the weak instrumental variable test are both statistically significant at the 1% level, indicating that *per capita* government health expenditures are endogenous and that the instrumental variable used in this paper is not weak.

**Table 4:** 2SLS regression results.

Variables	Model		
	All sample	Relative poverty households (*I_it_ ≤* 0.5807)	Ordinary households (*I_it_ >* 0.5807)
*Per capita* public health expenditure	0.2226^*^ (0.1333)	0.3087^*^ (0.1784)	0.1863 (0.1194)
Control variables	Yes	Yes	Yes
N	19,152	3,441	15,711
Endogeneity test	26.38^**^	14.52^***^	22.26^***^
Weak identification test	16.96^***^	12.45^***^	15.22^***^
The empirical *p*-value		0.086^*^	

(1) The endogenous test reports the *C* statistic (Chi^2^ value), and the weak instrumental variable test reports the Cragg–Donald Wald statistic (*F* value). (2) ^*^, ^**^ and ^***^ are statistically significant at the significance levels of 10%, 5% and 1%, respectively. (3) The empirical *p*-value is calculated using Fisher combination test (bootstrap 1,000 times).

## Conclusions and implications

### Conclusion

On the basis of the characteristics of relative poverty caused by household medical needs, this paper uses the China Family Panel Studies (CFPS) from 2016 to 2020 to identify relative poverty standard, analyzes the impact of public health expenditures on medical expenditures of different household types, and evaluates the heterogeneous effect of public health expenditures on ensuring the medical needs of relative poverty households. The panel threshold regression results show that 19.36% of the province’s *per capita* disposable income where the household is located is the identification standard of relative poverty households from the perspective of medical needs. Public health expenditures have a crowding-in effect on household medical expenditures and have a stronger impact on relative poverty households, an effect that is also confirmed by 2SLS regression. In addition, ensuring household medical needs through public health expenditures focuses on the level of basic medical needs, and the role of household healthcare expenditures that reflect high-level medical needs are not obvious.

### Implications and policy-making

In the future, to further improve the ability of households to cope with the risk of large rigid expenditures, and improve the efficiency of public health investment allocation, this paper proposes the following suggestions.

Firstly, a monitoring mechanism for relative poverty households should be established from the perspective of medical needs. There is no unified identification standard or measurement method for relative poverty households currently. For the formulation of the identification standard in this paper, the large rigid expenditures for medical and education by households are included in the analysis. The indicator is multidimensional and can be adjusted according to the household income and expenditure level. In the future, with continuous improvements in medical security policies, we can use the construction idea of this indicator to establish a dynamic monitoring mechanism for relative poverty households and include low-income and vulnerable groups with medical needs in the assistance and security system. This will improves the accuracy of the objective identification for relative poverty households.

Secondly, attention should be given to ensuring the basic medical needs of relative poverty households. According to Wagner’s Law, rigid expenditures can only continue to grow with social development. At present, relative poverty households have a higher vulnerability in maintaining large rigid household expenditures, and public health expenditures have a stronger crowding in effect on relative poverty households. This indicates that the basic medical needs of relative poverty households are still strong. Therefore, attention should be given to ensuring the basic medical needs of relative poverty households. The government needs to further increase investment in medical insurance, continuously improve the level of medical security, and reduce the medical burden of relative poverty households through transfer payments, medical assistance, special subsidies, etc. Avoid the phenomenon of returning to absolute poverty due to illness.

Thirdly, efforts should be made to clarify the heterogeneity among different types of households, provide targeted public health services. The conclusion indicates that the medical needs of relative poverty households and ordinary households are significant heterogeneous. The release effect of public health expenditures on relative poverty households’ medical needs is stronger, while the guarantee effect on publics’ high-level medical needs is not significant. The government can encourage social forces to intervene, improve the competitiveness of the public health service market, enrich the supply types of public health services, and meet the differentiated healthcare needs of different income groups.

## Data availability statement

Publicly available datasets were analyzed in this study. This data can be found here: [http://www.isss.pku.edu.cn/cfps/index.htm].

## Author contributions

ZL: conceptualization, methodology, and writing-original draft preparation. XY: formal analysis, data collection, and processing. ZZ: formal analysis and validation. YC: translation polishing, editing, writing-review, and supervision. All authors contributed to the article and approved the submitted version.

## Funding

This study was funded by the National Social Science Fund of China (No. CFA200250).

## Conflict of interest

The authors declare that the research was conducted in the absence of any commercial or financial relationships that could be construed as a potential conflict of interest.

## Publisher’s note

All claims expressed in this article are solely those of the authors and do not necessarily represent those of their affiliated organizations, or those of the publisher, the editors and the reviewers. Any product that may be evaluated in this article, or claim that may be made by its manufacturer, is not guaranteed or endorsed by the publisher.

## Footnotes

^1^The data comes from the CFPS database.

^2^At the Fourth Plenary Session of the 19th Central Committee of the Communist Party of China held on October, 2019, China announced a strategic change: in the future targeting relative poverty rather than absolute poverty which has been targeted since mid-1980s.

^3^The new medical reform has established the short-term goal of “effectively alleviating the ‘difficulty and cost of seeing a doctor’,” as well as the long-term goal of “establishing and improving the basic medical and health system that covers urban and rural residents, and providing safe, effective, convenient, and affordable medical and health services to the masses.”

^4^The main reason for selecting 1/3 of the *per capita* disposable income as the basis for delimitation is that currently, the World Bank regards social members whose income is lower than 1/3 of the average social income as relative poverty people. In addition, in some provinces in China that have already defined a relative poverty line, such as Zhejiang, Fujian, the identification standard is also basically based on 1/3 of the *per capita* income of households.

^5^This indicator mainly includes the self paid portion of household medical expenditures, excluding expenditures that have been reimbursed and are expected to be reimbursed, but includes the portion lent or paid by relatives and friends.

^6^The standard can also be expressed as 19.36% of the province’s *per capita* disposable where the household is located.
